# Supplementary material for: Epibrassinolide Regulates Lhcb5 Expression Though the Transcription Factor of MYBR17 in Maize
Source: Biomolecules. 2025 Jan 9;15(1):94. doi: 10.3390/biom15010094 (PMC11763093; doi:10.3390/biom15010094)
Supplement: Supplementary file 1 [file biomolecules-15-00094-s001.zip › biomolecules-3388327-supplementary.pdf]

Table S1 Fluorescence quantitative PCR primers

Species	Primer	Sequence（5'-3'）
Maize	β-Actin F	GATGATGCGCCAAGAGCTG
	β-Actin R	GCCTCATCACCTACGTAGGCAT
	Q-Lhcb3 F	GTGAACGTGCCGTCGTCTC
	Q-Lhcb3 R	GAACTCGCCCGTCAGGTAGC
	Q-Lhcb5 F	GCTCTTGCTCCATCCAAGATTC
	Q-Lhcb5 R	AGAGGGAGACGATCTTCTGAGC
	Q-Lhcb6 F	GCACCAGACCACCAGCTTCC
	Q-Lhcb6 R	ATGCTCTGGGGAACGCTCTT
	Q-MYBR17 F	GCCAGCCATGCACAGAAGTT
	Q-MYBR17 R	TCGCACTGTTCTCGCAAATC
	Q-JMJ14 F	CTCCTGTATGTGCGTCTGTTCC
	Q-JMJ14 R	CACTTTGTTCGCCATTTTCTCA
	Q-bHLH103 F	ACTACGCCAGTTTCTTCCACGA
	Q-bHLH103 R	CCGTGTAGTCCTGGAACGAGG
	Q-Sbp25 F	TTCAAGGGAACCAGCACAAAG
	Q-Sbp25 R	TGGATGACGGCTGAACTCG
	Q-bZIP76 F	AGAGCGTTGCAGAGGGATTG
	Q-bZIP76 R	AGCTTCCTGCTCAGGCTGTAGT
Arabidopsis thaliana	Actin2 F	ACGGAGACGGAGGACGAGAT
	Actin2 R	AATGTGAAGGCGAAGATCCAA
	Q-Lhcb5 F	GAGTAGTTAGGCTCTTTGGTGTTCAT
	Q-Lhcb5 R	GCGGTCAGGTGTTTTATGGG
	Q-MYBR17 F	AAAAGGGGTTCCATGGACGG
	Q- MYBR17 R	CGGGGATTTTGTGACGACGA

Table S2 Related transcription factors and target gene promoter amplification primers

Primer	sequence（5'-3’）
Lhcb3 F	CCCGGTCTTGACATTTCGG
Lhcb3 R	CCATTGCGCTGCTTCTGC
Lhcb5 F	TCCCTTTCAGGTTTCCTACTATTT
Lhcb5 R	CCATCGGAGCTGCCATT
Lhcb6 F	TCCGATGGGAGAAAGCC
Lhcb6 R	TGGTCAGTCAGAAGTGGGTT
MYBR17 F	ATGGCTAGGAAGTGCTCACACTGTGG
MYBR17 R	TCATGTTCCGATGGATGGGGC
bHLH103 F	ATGGACTCCTACAACTAC
bHLH103 R	TGCAGATTTGGCAAACCCA

Table S3 Five major enriched KEGG pathways in EBR vs CK (24 h)

Group	Pathway ID	Pathway	DEG number	Total number	*Pvalue*
BR vs CK	zma01110	Biosynthesis of secondary metabolites	181	1481	0.0004
	zma03010	Ribosome	97	504	7.14E-10
	zma00860	Porphyrin and chlorophyll metabolism	23	64	7.49E-07
	zma00195	Photosynthesis	26	101	2.43E-05
	zma00196	Photosynthesis antennaproteins	22	31	1.02E-10

Table S4 Correlation coefficients of selected transcription factors and Lhcb3 Lhcb5 Lhcb6

	Lhcb3	Lhcb5	Lhcb6	MYBR17	Jmj14	bHLH103	Sbp25	bZIP76
Lhcb3	1.00							
Lhcb5	0.57	1.00						
Lhcb6	0.86	0.88	1.00					
MYBR17	0.66	0.41	0.63	1.00				
Jmj14	-0.34	-0.92	-0.67	-0.15	1.00			
bHLH103	0.68	0.83	0.85	0.49	-0.76	1.00		
Sbp25	0.26	-0.53	-0.18	-0.05	0.55	-0.15	1.00	
bZIP76	-0.71	-0.31	-0.44	0.20	0.27	-0.48	-0.29	1.00

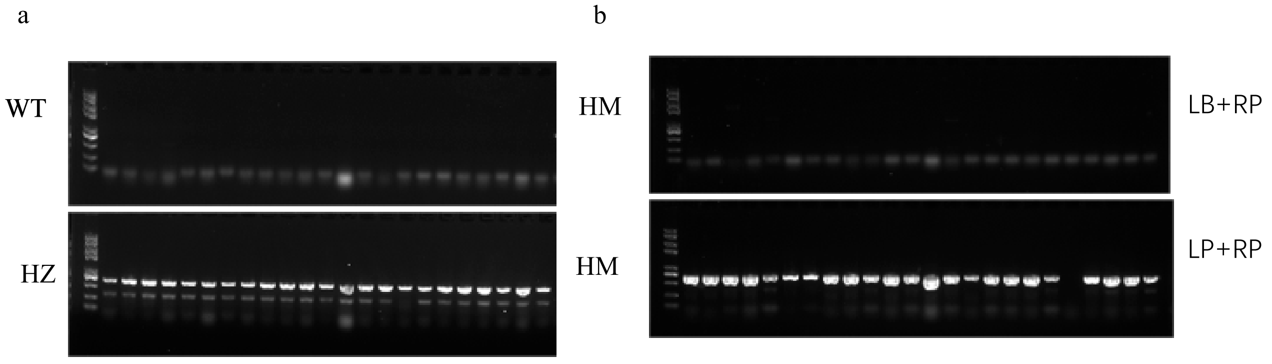

Figure S1 Identification results of Arabidopsis thaliana ' three primer method '

(a) electrophoresis results of mutant LP + RP and LP + LB. (b) electrophoresis results of heterozygous and wild type LP + RP and LP + LB
